# Detection and dynamics of circulating tumor cells in patients with high-risk prostate cancer treated with radiotherapy and hormones: a prospective phase II study

**DOI:** 10.1186/s13014-020-01577-5

**Published:** 2020-06-01

**Authors:** Almudena Zapatero, Antonio Gómez-Caamaño, María Ángeles Cabeza Rodriguez, Laura Muinelo-Romay, Carmen Martin de Vidales, Alicia Abalo, Patricia Calvo Crespo, Luis Leon Mateos, Carlos Olivier, Lorena Vega Vega Piris

**Affiliations:** 1 Radiation Oncology Department, Hospital Universitario de la Princesa, Health Research Institute IIS-IP, Diego de León 62, 28006 Madrid, Spain; 2 Hospital Universitario de Santiago de Compostela, Santiago de Compostela, Spain; 3 Hospital Universitario 12 de Octubre, Madrid, Spain; 4 Liquid Biopsy Analysis Unit, Health Research Institute of Santiago (IDIS), CIBERONC, Santiago de Compostela, Spain; 5 Methodology Unit, Health Research Institute of Hospital Universitario de La Princesa, Madrid, Spain

**Keywords:** Prostate cancer, Circulating tumor cells (CTCs), Radiotherapy, Androgen suppression, Treatment outcome, Prognostic factor, detection

## Abstract

**Background:**

Circulating tumor cells (CTCs) are an established prognostic marker in castration-resistant prostate cancer but have received little attention in localized high-risk disease. We studied the detection rate of CTCs in patients with high-risk prostate cancer before and after androgen deprivation therapy and radiotherapy to assess its value as a prognostic and monitoring marker.

**Patients and methods:**

We performed a prospective analysis of CTCs in the peripheral blood of 65 treatment-naïve patients with high-risk prostate cancer. EpCAM-positive CTCs were enumerated using the CELLSEARCH system at 4 timepoints. A cut off of 0 vs ≥ 1 CTC/7.5 ml blood was defined as a threshold for negative versus positive CTCs status.

**Results:**

CTCs were detected in 5/65 patients (7.5%) at diagnosis, 8/62 (12.9%) following neoadjuvant androgen deprivation and 11/59 (18.6%) at the end of radiotherapy, with a median CTC count/7.5 ml of 1 (range, 1–136). Only 1 patient presented a positive CTC result 9 months after radiotherapy. Positive CTC status (at any timepoint) was not significantly associated with any clinical or pathologic factors. However, when we analyzed variations in CTC patterns following treatment, we observed a significant association between conversion of CTCs and stages T3 (*P* = 0.044) and N1 (*P* = 0.002). Detection of CTCs was not significantly associated with overall survival (*P* > 0.40).

**Conclusions:**

Our study showed a low detection rate for CTCs in patients with locally advanced high-risk prostate cancer. The finding of a de novo positive CTC count after androgen deprivation therapy is probably due to a passive mechanism associated with the destruction of the tumor. Further studies with larger samples and based on more accurate detection of CTCs are needed to determine the potential prognostic and therapeutic value of this approach in non-metastatic prostate cancer.

**Trial registration:**

ClinicalTrials.gov ID: NCT01800058.

## Background

High-risk prostate cancer is a challenging disease. The combination of high-dose radiotherapy and androgen deprivation represents the standard of care and has yielded encouraging results [1]. More recently, preliminary results with the addition of abiraterone acetate or docetaxel [2, 3] to luteinizing hormone–releasing hormone agonists point to an improved outcome in selected patients. However, given that some subgroups of patients with unfavorable prognostic factors do not respond as well to these treatments, it is necessary to identify improved biomarkers in order to identify patients who require a more aggressive approach. Growing evidence of the clinical importance of detecting circulating tumor cells (CTCs) in the peripheral blood of cancer patients indicates that this is a relevant prognostic and predictive biomarker after care [4, 5].

Counting CTCs using the CELLSEARCH assay (Menarini, Silicon Biosystems Inc., Bologna, Italy) was recently proven to be a reliable prognostic biomarker in metastatic castration-resistant prostate cancer [6–9]. Even more recently, the results of 2 prospective trials with abiraterone and chemotherapy [10] showed that changes in CTCs as early as 4 weeks after treatment identified patients who were not benefiting from treatment, thus suggesting that the CTC count could be an intermediate biomarker for overall survival in advanced metastatic prostate cancer.

These data contradict the limited available information in non-metastatic prostate cancer. The few studies that have examined the response of CTCs to radiotherapy or radical prostatectomy in patients with localized prostate cancer have reported conflicting results [11, 12]. Consequently, we conducted a prospective multicenter phase II study to determine the proportion of patients with high-risk or locally advanced prostate cancer who presented CTCs in peripheral blood at diagnosis and to assess the association between this finding and clinically recognized prognostic factors. We also analyzed the patterns of change in CTCs following androgen deprivation and radiotherapy in order to assess their potential impact on clinical outcome. We hypothesized that the CTC count could not only identify patients with a poorer prognosis, but also determine the degree of response to treatment (sensitivity to radiotherapy and hormone therapy) in patients with high-risk prostate cancer.

## Material and methods

### Patients and treatment

The study population comprised 66 treatment-naïve patients with non-metastatic prostate cancer recruited from 3 Spanish teaching hospitals between 2014 and 2016. Of these, 65 were eligible for the CTC analysis. Patients aged 18 years or older with histologically confirmed adenocarcinoma of the prostate and ≥ 1 high-risk factors (prostate-specific antigen [PSA] > 20 ng/ml, Gleason 8–10, stage T3–4), N0 or N1, a Karnofsky performance score of ≥70, and a life expectancy of more than 5 years were eligible for inclusion. The study was approved by the independent review board at each participating center and conducted according to the provisions of the Declaration of Helsinki and the Good Clinical Practice Guidelines of the International Conference on Harmonization. Patients provided their written informed consent before participating in the study.

Patients received combined modality therapy comprising high-dose radiotherapy and hormone therapy. Radiotherapy was administered using 3-dimensional conformational radiation therapy (45 patients) or intensity-modulated radiation therapy (20 patients) at a median dose of 76.3 Gy (range, 74.8–79.2) and 81.7 Gy (range, 70.6–82-8), respectively. Hormone therapy consisted of 4 months of neoadjuvant and concomitant androgen deprivation followed by adjuvant androgen deprivation for a median of 26 (17–36) months.

### Procedures

**Fig. 1:**
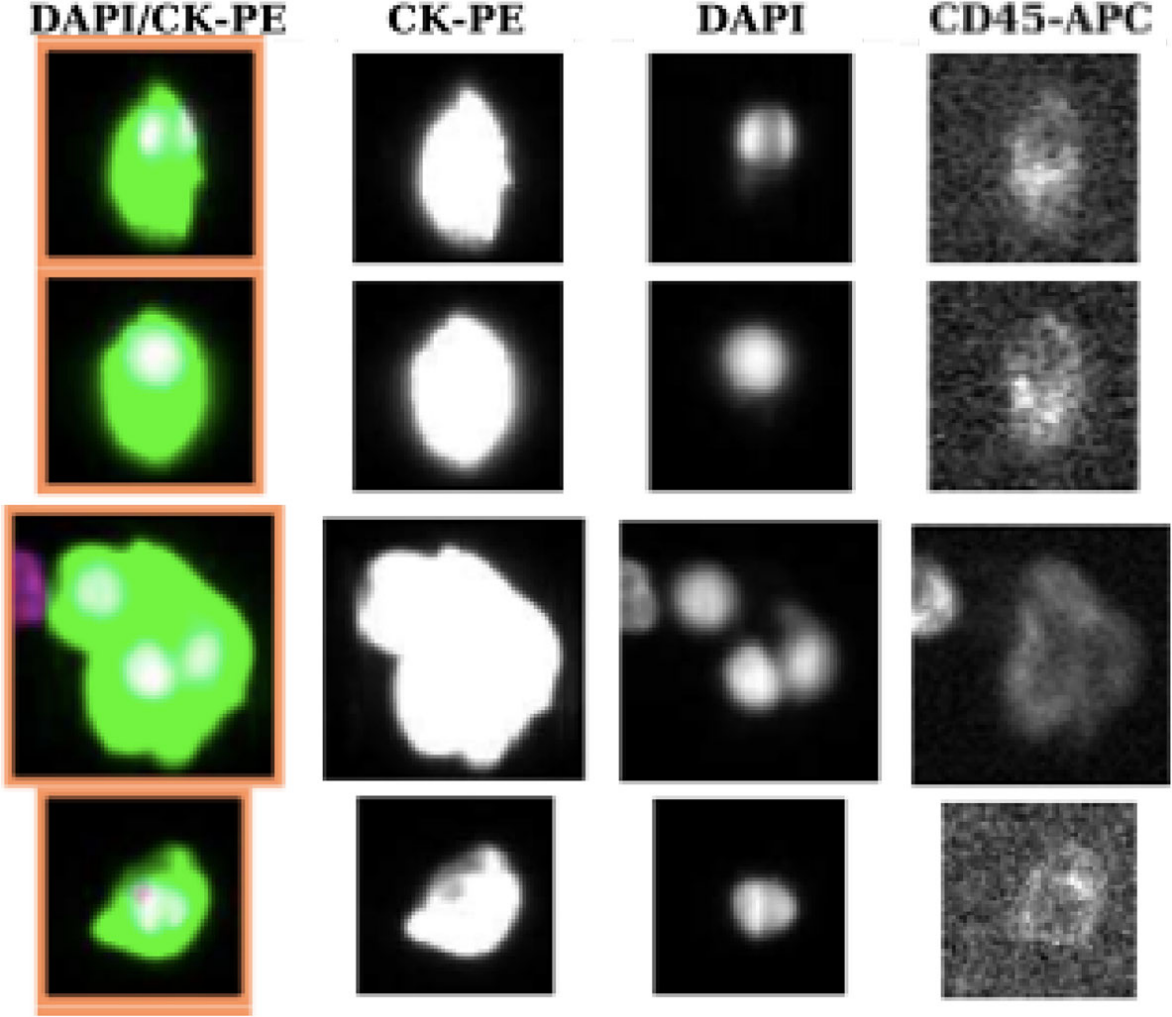
Images of CTCs obtained from a patient with prostate cancer using the CELLSEARCH system. Only round-oval, DAPI^+^, CD45^−^, and CK^+^ cells were considered CTCs

Peripheral blood samples were collected prospectively and analyzed to detect CTCs at 4 timepoints: 1) baseline or before starting treatment (visit 1); 2) after neoadjuvant androgen deprivation and before radiotherapy (visit 2); 3) at the end of radiotherapy (visit 3); and 4) 6–12 months after the end of radiotherapy in those patients with 0 CTCs in the first determination and conversion to positive CTCs in the following determinations (visit 4). One tube (7.5 mL) of peripheral blood was obtained from each patient at each sampling point. We used the CELLSEARCH assay (Menarini, Silicon Biosystems Inc., Bologna, Italy), which is the only CTC detection method approved by the United States Food and Drug Administration. Therefore, peripheral blood was analyzed using the CELLSEARCH Circulating Tumor Cell kit at the Liquid Biopsy Analysis Unit of the Health Research Institute of Santiago de Compostela. The system immunoisolated cells that were positive for epithelial cell adhesion molecule (EpCAM). Enriched cells were then labelled with phycoerythrin-conjugated anti-cytokeratins antibodies, allophycocyanin-conjugated anti-CD45 antibodies, and 4,6-diamino-2-phenylindole to identify the nucleus. Digital images of the 3 different fluorescent dyes were acquired using the CELLTRACKS Analyzer and a 12-bit camera. The images were reviewed by trained operators in order to determine the CTC count (Fig. 1). Round-oval, DAPI^+^, CD45^−^, and CK^+^ cells were considered CTCs.

### Statistical methods

The frequency of expression of CTCs was estimated with its 95% confidence interval (CI). Initially a cutoff point of ≥3 CTCs/7.5 mL of blood was taken as the reference baseline. Because of the low CTC counts observed and for statistical purposes, we empirically established a cut-off of 0 vs ≥1 CTCs/7.5 mL blood as the thresholds between negative and positive CTC status. The association between the presence and development of CTCs over time and well-known clinicopathological features was assessed using the χ^2^ test or Fisher exact test. Overall survival, metastasis-free survival, and biochemical failure–free survival were estimated using the Kaplan-Meier method. The curves were compared using the log-rank test. All events were defined as time until death from any cause (OS), distant metastasis (MFS), biochemical failure (bFS), or date of last follow-up visit. Cox regression models were constructed to estimate the hazard ratio and 95% CI as a measure of the effect and were adjusted for potential confounding variables (*P* ≤ 0.20)..All hypothesis tests were performed using 2-tailed alternatives. Statistical significance was set at *P* < 0.05.

## Results

The initial study sample comprised 66 high-risk patients, of whom 65 were evaluable for the analysis (1 patient was lost to follow-up before completing the CTC study). The median age was 71 years (range, 53–79), and the median pretreatment PSA value was 12.6 ng/mL (range, 3.2–68.7). The Gleason score was ≥8 in 28 patients, and 48 patients were in clinical stage T3. The median interquartile range (IQR) follow-up was 55 (23–64) months. Patient and treatment characteristics are summarized in Table 1.

**Table 1:** Summary of patient and treatment characteristics

	***N*** = 65
Median (range) age, years	71 (53–79)
> 65	16 (25%)
≤ 65	49 (75%)
Median follow-up, months (range)	55 (10–40)
Median PSA, ng/mL (range)	12.6 (3.2–68.7)
< 10	22 (34%)
10–20	20 (31%)
> 20	23 (35%)
Gleason Group 1	5 (8%)
Group 2	13 (20%)
Group 3	19 (29%)
Group 4	20 (31%)
Group 5	8 (12%)
T stage T1	1 (2%)
T2	17 (26%)
T3	47 (72%)
Number of high-risk factors 1	35 (54%)
2	28 (43%)
3	2 (3%)
N stage N0	52 (80%)
N1	13 (20%)
Technique 3DCRT	44 (68%)
Technique IMRT	21 (32%)
Median dose 3DCRT, Gy (range)	76.3 (74.78–79.16)
Median dose IMRT Gy (range)	81.7 (70.6–82-8)

*PSA* prostate-specific antigen, *3DCRT* 3-dimensional conformal radiation therapy, *IMRT* intensity-modulated radiation therapy

**Fig. 2:**
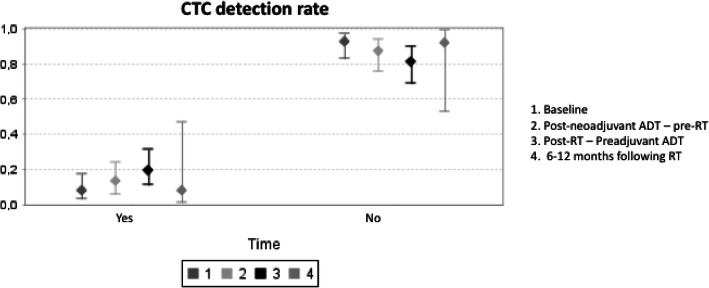
CTCs detection rate at 4 timepoints: 1) baseline; 2) after neoadjuvant androgen deprivation therapy; 3) at the end of radiotherapy; and 4) 6–12 months after the end of radiotherapy in those patients with 0 CTCs in the first determination and a change to positive CTCs in the following determinations. CTC, circulating tumor cell; ADT, androgen deprivation therapy

The median CTC count/7.5 mL at all of the timepoints was 1 (1–136). The changing pattern of detection over time is summarized in Table 2 and Fig. 2. At diagnosis (visit 1), CTCs were detected in only 5/65 patients (7.5%). Of these, 3 became negative at visit 2 (following neoadjuvant androgen deprivation), and the remaining 2 patients became negative following radiotherapy (visit 3). At visit 2, CTCs were analyzed in 62 patients (3 patients did not complete the analysis), and de novo CTC positivity was detected in 6 patients whose values became negative at visits 3 and 4. We also observed de novo CTC positivity at visit 3 (end of radiotherapy) in 8 patients. Only 1 patient presented positive CTC results 9 months after radiotherapy.

**Table 2:** CTC counts before and after treatment

CTC Timing	CTC Positivity/Determinations
CTC1	
≥1 cells/7.5 ml (%) (range)	5/65 (7.5%) (1-6)
0 cells /7.5 ml	60
CTC2	
≥1 cells/7.5 ml (range)	8/62(12.9%) (1-1) *New presentation in 6*
0 cells /7.5 ml	54
Not performed	3
CTC3	
≥1 cells/7.5 ml (range)	11/59 (18.6%) (1-136) *New presentation in 10*
0 cells /7.5 ml	48
Not performed	6
CTC4	
≥1 cells/7.5 ml (range)	1/13 (7.6%) (1)
0 cells /7.5 ml	*No new: persistence from CTC3* 12

CTC 1: Baseline; CTC 2: After neoadjuvant androgen deprivation therapy and before radiotherapy; CTC 3: After radiotherapy; CTC 4: 6-12 months after radiotherapy in patients with new positivity at CTC 2 or CTC 3

Positive CTC status (at any timepoint) was not significantly associated with clinical and pathologic factors (patient age, pre-treatment PSA, T stage, N stage, Gleason score, and number of positive cores). Similarly, we did not find an association between positive CTC status and the presence of secondary tumors (Table 3). However, when we analyzed the variation pattern of positive CTC results following treatment, we observed a significant association between CTC conversion and stages T3 (*P* = 0.044) and N1 (*P* = 0.002).

**Table 3:** CTC positivity and association with prognostic factors

	CTC1+	CTC2+	CTC3+	CTC4+
Age, y ≤65	1	2	2	0
>65	4	6	9	1
*p* value	1.000	1.000	0.712	1.000
PSA <10 ng/mL	2	1	4	1
10-20 ng/mL	2	5	4	0
>20 ng/mL	1	2	2	0
*p* value	0.852	0.128	0.711	1.000
Gleason <8	3	7	5	1
sum ≥ 8	2	1	6	0
*p* value	1.000	0.128	0.502	1.000
T Stage				
<T3	0	1	6	0
≥T3	5	7	5	1
*p* value	0.312	0.668	0.062	1.000
N Stage				
0	5	7	7	1
1	0	1	4	-
*p* value	0.574	1.000	0.053	
Secondary tumors				
Yes	3	6	9	0
No	2	2	2	1
*p* value	0.621	1.000	0.484	0.231

*PSA* prostate-specific antigen

Only 1 patient experienced biochemical relapse and metastatic disease (at 31 months) and is currently alive (no positive CTCs at any timepoint). Six patients died, none of them from prostate cancer. Detection of CTCs at diagnosis and following treatment was not significantly associated with overall survival (*P* = 0.40).

## Discussion

The main objective of the present study was to assess the viability of detecting CTCs in non-metastatic high-risk prostate cancer patients. Our findings revealed a low count and incidence of CTCs. Only 5 out of 65 patients (7.5%) harbored CTCs at diagnosis, that is, at the bottom range of the detection rates of other studies. The few published reports using the CELLSEARCH system in patients with localized prostate cancer showed positivity rates of 5–27% [13–15]. These studies were carried out in patients treated predominantly with radical prostatectomy or brachytherapy and included early stages of prostate cancer. In an attempt to improve the CTC detection rate, we focused the design of the study on including only patients with locally advanced, high-risk disease (13 patients had N1 disease and 30 patients had 2 or more risk factors). To our knowledge, this is the first prospective study performed in this subgroup of patients treated homogeneously with high-dose radiotherapy plus androgen deprivation and with repeated CTC determinations at predetermined intervals to monitor treatment response.

The many methods used to detect CTCs range from real-time polymerase chain reaction to cell size–based separation or immunomagnetic beads conjugated with anti-EpCAM antibodies [16–19], each of which is subject to intrinsic limitations, including reproducibility. For metastatic prostate cancer, the CELLSEARCH system was shown to provide prognostic information in several large clinical trials [7, 8, 20, 21]. Nevertheless, in localized prostate cancer, the CELLSEARCH methodology is now believed to underestimate the actual number of CTCs, perhaps owing to fragmentation of conventional CTCs and the inability to detect the less epithelial CTCs [13]. Data from recent studies have shown that EpCAM-based enrichment alone could not detect all CTC subpopulations [22].

Several attempts have been made to overcome this limitation. Theil et al. [23] used a new system, CellCollector to isolate in vivo CTCs from patients with different stages of prostate cancer and found more frequent detection of CTCs than with the CELLSEARCH system. Kuske et al. [24] also reported improved detection of CTCs in nonmetastatic prostate cancer patients by combining 3 independent CTC assays: the CELLSEARCH system, CellCollector, and EPISPOT (an EpCAM-independent enrichment method). Peripheral blood samples were screened for CTCs before radical prostatectomy in 86 high-risk prostate cancer patients and 3 months after radical prostatectomy in 52 patients. The cumulative positivity rate of all 3 CTC assays was 81.3% (87/107), with 21.5 (23/107) of patients harboring ≥5 CTCs per blood sample. The authors hypothesize that the correlation observed with established risk factors and the persistence of CTCs 3 months after surgery could suggest the potential clinical relevance of CTCs as markers of minimal residual disease in prostate cancer.

The second objective of the present study was to analyse whether the variation in the CTC count at predetermined intervals during the treatment regimen would predict outcome and enable a real-time response. We expected a decline in the numbers of CTCs following treatment. Unpredictably, an increasing and transient CTC count was detected de novo after androgen deprivation and radiotherapy. The CTC detection rate was 7.5% at diagnosis, which increased to 12.9% following neoadjuvant androgen deprivation and 18.6% at the end of radiotherapy before decreasing again to 7.5% at 9 months after the end of radiotherapy. When we investigated this changing pattern of CTC positivation more specifically, we observed a significant association with locally advanced disease (T3–4 stage, *p* = 0.044; and N1 stage, *p* = 0.002). Although we do not have a clear explanation for this finding, we believe that it could be due to a passive mechanism associated with the destruction of the tumor. A further analysis with longer follow-up is required to clearly determine the relevance of CTC positivation during the treatment.

Importantly, other authors have reported similar findings. Stott et al. [25] observed that of 11 patients with preoperative CTCs counts below the cutoff, 4 had transient elevations in CTCs during the follow-up period. Tsumura et al. [12] evaluated whether prostate brachytherapy procedures had a potential risk for hematogenous spillage of prostate cancer cells in 59 patients using the CELLSEARCH system. The authors detected CTCs from samples immediately after the brachytherapy procedure in 7 patients (intraoperative CTC detection rates were significantly higher than preoperative ones), although they did not repeat the CTC analysis during follow-up to evaluate whether those CTCs could actually survive and proliferate at distant sites.

We were unable to show a significant association between positive CTC status at diagnosis and known clinical and histologic prognostic factors. This lack of correlation has been extensively observed in studies carried out in localized prostate cancer [11, 16, 25]. The small sample size and the low CTC detection rate might limit the relevance of these results.

In one of the longest prospective series (152 patients analysed using the CELLSEARCH platform), Meyer et al. [26] did not observe a significant relationship with PSA, Gleason score, or pT stage. The CTC detection rate in their series was only 11%. Pal et al. [27] used a modified isolation procedure on the CELLSEARCH platform in 35 patients with high-risk, localized prostate cancer. With a CTC detection rate of 49% prior to surgery, they did not observe any correlation between the presence of CTCs and clinicopathological prognostic features. In contrast, Kuske et al. [24] used the EPISPOT method before radical prostatectomy and found a significant association between CTCs and PSA and clinical T stage. The detection rate with EPISPOT was 58.7%. However, they failed to show any clinical correlation with the other assays (the CELLSEARCH system and the in vivo CellCollector).

Only 1 patient in our series experienced biochemical and distant failure, and 6 patients have since died, none of them from prostate cancer. Again, we did not observe any association between CTC count and overall survival. Although some authors report a trend towards shorter recurrence times [28], no studies to date have proved the predictive value of CTCs in disease-free or overall survival.

Our study is subject to the limitations inherent to an observational feasibility study, namely, the small sample size and short follow-up. The main strengths are its prospective design with pre-established CTC determinations at predetermined intervals to monitor treatment response.

In conclusion, the data obtained from this study showed a low detection rate for CTCs in patients with high-risk prostate cancer. The finding of de novo positive CTC counts after androgen deprivation and radiotherapy (mostly with very low levels and not maintained over time) may be due to a passive mechanism associated with destruction of the tumor. Further studies with a longer follow-up period, larger samples with more events and a more acute assessment method are needed to determine the potential prognostic and therapeutic value of detection of CTCs in nonmetastatic prostate cancer.

## Data Availability

The datasets used and/or analysed during the current study are available from the corresponding author on reasonable request.

## Abbreviations

CTCs: Circulating tumor cells
CI: Confidence interval
Gy: Gray
IMRT: Intensity-modulated radiation therapy
IQR: Interquartile range
PSA: Prostatic specific antigen
3DCRT: 3-dimensional conformal radiation therapy
